# Lifestyle risk factor related disparities in oral cancer examination in the U.S: a population-based cross-sectional study

**DOI:** 10.1186/s12889-020-8247-2

**Published:** 2020-01-31

**Authors:** Aderonke A. Akinkugbe, Dina T. Garcia, Tegwyn H. Brickhouse, Maghboeba Mosavel

**Affiliations:** 1 Department of Dental Public Health and Policy, School of Dentistry, Virginia Commonwealth University, 1101 East Leigh Street, Richmond, VA 23298-0566 USA; 2 Institute for Inclusion, Inquiry, and Innovation, Virginia Commonwealth University, Richmond, VA USA; 3 Department of Health Behavior and Policy, School of Medicine, Virginia Commonwealth University, Richmond, VA USA

**Keywords:** Oral cancer examination, Cancer screening, Risk factors, Smoking, Alcohol consumption, Health disparities

## Abstract

**Background:**

Oral cancers account for 3% of annual U.S. cancer diagnosis, 2 in 5 of which are diagnosed late when prognosis is poor. The purpose of this study was to report the population-level prevalence of oral cancer examination among adult smokers and alcohol drinkers and assess if these modifiable lifestyle factors are associated with receiving an oral cancer examination.

**Methods:**

Adult participants ≥30 years (*n* = 9374) of the 2013–2016 cycles of the National Health and Nutrition Examination Survey were included. Oral cancer examination (yes/no), smoking (never, former, current) and alcohol use (abstainers, former, current) were self-reported. Survey-logistic regression estimated odds ratios (OR) and 95% confidence intervals (CIs) of ever and past year oral cancer examination adjusted for age, gender, race/ethnicity, education, income, and time since last dental visit.

**Results:**

One third (33%) reported ever been examined for oral cancer, 66% of whom reported an examination in the past year. Adjusted OR (95% CI) of past year examination comparing current and former smokers to non-smokers were 0.51 (0.29, 0.88) and 0.74 (0.53, 1.04) respectively. Similarly, current and former alcohol drinkers relative to abstainers were less likely to report a past year oral cancer examination, OR (95% CI) = 0.84 (0.53, 1.30) and 0.50 (0.30, 0.83) respectively.

**Conclusion:**

This study showed that smokers and alcohol users were less likely than abstainers to self-report a past year oral cancer examination. Access to affordable and targeted oral cancer examination within the dental care setting might ensure that these high-risk individuals get timely examinations and earlier diagnosis that might improve prognosis and survival.

## Background

Cancer is a major public health problem worldwide and the second leading cause of death in the United States [1]. In 2016, an estimated 370,309 people in the U.S. were living with head and neck cancers. With an estimated 53,000 new cases for 2019 and 10,860 expected deaths, head and neck cancers represent 3% of all cancer cases in the U.S [2].

Etiology of head and neck cancers is complex, involving a multistage development process and several genetic and environmental factors. Oropharyngeal cancers, a subset of head and neck cancer has the human papillomavirus (HPV) as an important risk factor [3] while modifiable lifestyle behaviors including smoking and alcohol consumption are implicated in the etiology of oral cavity cancers. In a pooled analysis of 15 case control studies, smoking was associated with a 2-fold higher odds of oral cavity cancers among never drinkers of alcohol and excessive alcohol consumption was associated with increased odds of oral cancers among never smokers [4]. Furthermore, dual use of tobacco products and alcohol act synergistically and together accounts for 3 in 4 oral cavity cancer cases [5].

While U.S. rates of oral cavity cancers have steadily declined over the past few decades consistent with successful public health efforts that have resulted in declines in smoking and alcohol consumption, rates of new oropharyngeal cancer cases have been on the rise at an average rate increase of 0.8% per year over the last decade [2]. This increase in prevalence is attributable to the rising HPV infection associated head and neck cancers [3, 6, 7]. Despite known risk factors for oral cavity cancers and a cancer site that is readily accessible, 2 in 5 cases are diagnosed late when prognosis is poor [8]. Survival rates for oral cancers are stage dependent, with an 84% relative 5-year survival for localized cancers and 64 and 39% respectively for regional and distant metastases. Relative survival also vary based on race/ethnicity and is estimated to range from 48 to 66% [1].

Screening programs (population-based, opportunistic among those attending care for other reasons or targeted to certain high risk groups) [9] are in place for several major cancers including colon and breast cancers and have been instrumental in early detection. Oral cancers are also amenable to screening and risk modification. Screening test for oral cancers is a systematic clinical examination of the oral cavity and includes a visual inspection of the face, neck, lips, labial mucosa, buccal mucosa, gingiva, floor of the mouth, tongue, and palate as well as palpating the regional lymph nodes. Any abnormality lasting for more than 2 weeks is reevaluated and considered for a biopsy [10]. Screening is thus poised to identify precancerous lesions and potentially malignant oral lesions with the potential to improve outcomes including survival [11]. Nonetheless, evidence of oral cancer screening effectiveness is grossly limited [3, 9, 12–14]. Indeed, a Cochrane systematic review, found no meaningful difference in incidence rates between screened and control group, but reported a statistical significant 24% reduction in oral cancer mortality for screened high risk individuals who used tobacco, alcohol or both when compared to the control group [9] based on findings from a cluster randomized study in India [12, 14]. The American Cancer Society has no routine screening test recommendation guidelines for head and neck cancers [15], and the American Dental Association (ADA) likewise, found insufficient evidence to determine if screening alters disease-specific mortality in asymptomatic people seeking dental care. The ADA, however, recommends that dental providers remain alert for signs of potentially malignant lesions or early-stage cancer in patients during routine oral examinations, particularly in those who use tobacco products or consume large amounts of alcohol [16].

Therefore, this study seeks to report the population-level prevalence of oral cancer examination among adult smokers and alcohol drinkers in the U.S. and assess if these modifiable lifestyle factors are associated with ever and past year receipt of an oral cancer examination. This study expands the current literature [26] by assessing the individual and joint effects of alcohol and cigarette use on receiving oral cancer examination among U.S. adults using the most recent nationally representative data. Findings from this study can inform future public health efforts to enhance timely administration of oral cancer screenings among high-risk groups.

## Methods

### Data source, study design and population

Data for this cross-sectional study comes from the 2013–2014 and the 2015–2016 cycles of the National Health and Nutrition Examination Survey (NHANES). The NHANES is a stratified, multistage, probability sampling survey conducted by the National Center for Health Statistics of the Centers for Disease Control and Prevention [17]. Participants (*n* = 9374), 30 years and older, who completed socio-demographic and oral health questionnaires were included in this study. The current study is a human observational study and adhered to the Strengthening the Reporting of Observational studies in Epidemiology (STROBE) guidelines. The 2 cycles of NHANES (2013–2014 and 2015–2016) used for this study represent the most recent nationally representative data on OC examination that was collected after the ADA’s 2010 recommendation that dental providers remain alert for signs of potentially malignant lesions or early-stage cancer in patients during routine oral examinations, particularly in those who use tobacco products or consume large amounts of alcohol.

### Exposures

Two lifestyle-related and modifiable risk factors for oral cavity cancer were investigated as exposures.

#### Smoking

Current cigarette use among NHANES participants was based on self-reported lifetime use of 100 cigarettes and current cigarette use status. Participants who responded ‘yes’ to having smoked 100-lifetime cigarettes and ‘yes’ to current smoking were categorized as *current smokers*; those who responded ‘yes’ to using 100 cigarettes in their lifetime but ‘no’ to current smoking were categorized as *former smokers*. Those who have not used 100-lifetime cigarettes were categorized as *non-smokers* [18, 19].

#### Alcohol consumption

Alcohol consumption among NHANES participants was based on the quantity and frequency of alcohol use in the past 12 months. Category of alcohol use was created based on previously used thresholds for the NHANES study [20]. *Lifetime abstainers* were those who reported ever consuming less than 12 alcoholic drinks; *former drinkers* were those who had consumed > 12 lifetime drinks but none during the past year; current alcohol use was sub-categorized into non-excessive current drinkers and excessive current drinkers. *Non-excessive current drinkers* were those who consume on average 14 drinks/week for men or 7 drinks/week for women without having consumed ≥5 drinks in a single day within the past year while *excessive current drinkers* were those who consumed > 14 standard drinks/week for men or > 7 standard drinks/week for women OR reported consuming ≥5 drinks on ≥1 days in the past year. There was no meaningful difference in regression estimates for current non-excessive and excessive alcohol users and were thus combined into a single current use group in multivariable analysis. The specific NHANES questionnaires for smoking and alcohol use are available for review in the online appendix.

### Outcome

#### Oral cancer examination and time since last examination

Questions about oral cancer examination were asked of participants at least 30 years old. In both survey cycles, oral cancer examination was based on the question: “Have you ever had an exam for oral cancer in which the doctor or dentist pulls your tongue, sometimes with gauze wrapped around it, and feels under the tongue and inside the cheeks?” Responses were yes or no. In the 2013–2014 survey year, an additional question: “Have you had an exam for oral cancer in which the doctor or dentist feels your neck?” was asked of participants. Responses were yes or no. Of the 950 participants in the 2013–2014 survey year who reported a neck palpation examination, 650 of them reported also receiving an oral examination and 300 received only a neck palpation examination. Excluding these 300 individuals from data analysis did not meaningfully change the results, thus they were retained in all analysis.

In both survey cycles, participants who responded ‘yes’ to having had an oral cancer examination were asked to report when they had the most recent oral cancer examination (within the past year, 1–3 years ago and > 3 years ago) and the type of professional that performed the examination (Doctor/physician, Nurse/nurse practitioner, Dentists including oral surgeons, Dental Hygienists and other).

### Covariates

Because the question on oral cancer examination was asked only of those 30 years and older, we restricted our study sample to participants who were at least 30 years old. Age in years was categorized into 30–65 and ≥ 65 years for descriptive purposes and modeled as continuous in logistic regression; gender was reported as male or female; race/ethnicity was categorized into non-Hispanic whites, non-Hispanic blacks, Mexican American, other Hispanics and Other. Education was categorized into: ≤high school, some college, college or more while household income was categorized into <$20,000/year, $20–45,000/year and > $45,000/year. Time since last dental visit was categorized into < 1 and ≥ 1 year.

### Missing data

While a large proportion (25%) were missing alcohol use information, an exploration of the pattern of missing data for the covariates used in this investigation indicated that the majority of missing alcohol use information was not dependent on the missingness of other covariates, thus suggesting that alcohol use data are likely missing completely at random (MCAR) as opposed to missing at random (MAR) which is missing given observed covariates. Hence, we expect that the findings from the complete case analysis we conducted will be similar to the findings we would have obtained had there been no missing alcohol use information.

### Statistical analysis

Data analysis was restricted to participants with no missing information on all covariates, the outcome (oral cancer examination) and exposures (smoking and alcohol use). This corresponds to a sample size of (*n* = 8781) for the analysis with smoking as the main exposure; and a sample size of (*n* = 6586) for the analysis with alcohol use as the main exposure, out of the 9374 eligible participants. Data analysis began with an overall distribution of socio-demographic factors for the study population as well as a distribution of these factors according to time since last dental visit and self-report of ever receiving an oral cancer examination. Weighted percentages and standard errors were reported, and large sample Wald tests assessed differences between groups. Next the distribution of clinical measures (last dental visit, reason for dental visit, and type of professional) was assessed according to time since the last oral cancer examination, restricted to those who have ever received an examination. Adjusted for confounders, survey-logistic regression estimated odds ratios (ORs) and 95% confidence intervals (CIs) for the independent associations between smoking and alcohol consumption with self-report of *ever* receiving an oral cancer examination and separately between smoking and alcohol with self-reported *past year* receipt of an oral cancer examination. Given previous reports indicating that dual use of tobacco and alcohol use act synergistically to increase the risk for oral cancers [5], we assessed if self-report of ever or past year receipt of oral cancer examination was greater among dual users than among smokers alone or alcohol users alone. We accomplished this by creating a composite variable of smoking and alcohol consumption: dual use (current use of both cigarettes and alcohol); smoking only (current use of cigarettes); alcohol use only (non-excessive, and excessive current users of alcohol combined); neither (abstainers from alcohol and non-smokers including former smokers and former alcohol users).

To minimize bias likely to result from a higher likelihood to report an oral cancer examination in those who have seen a healthcare (dentist or otherwise) provider, we conducted a sensitivity analysis restricted to participants who reported a past year dental visit (because dental care providers provided the most oral cancer examinations in this population) and used covariate adjusted survey-logistic regression to estimate associations between smoking and alcohol consumption with self-reported ever and past year receipt of an oral cancer examination. Statistical tests were 2-sided and *p*-values < 0.05 were considered statistically significant. Data analyses were conducted in SAS v. 9.4 (SAS Institute, Cary NC), accounted for the complex survey and sampling design of NHANES with degrees of freedom calculated by SAS. Subpopulation analysis adhered to the guidelines provides by NHANES, available at https://wwwn.cdc.gov/nchs/data/nhanes/2011 2012/analyticguidelines/analytic_guidelines_11_16.pdf

## Results

A majority (76%) of the study participants were aged 30–65 years and about half were female (53%). Non-Hispanic white participants comprised 68% of the sample and 11% were non-Hispanic blacks. Educational attainment was approximately equally distributed among participants at 33% for ≤high school, 31% for some college and 33% for ≥college degree. About half were non-smokers (54%) and 58% were current alcohol consumers. Overall, 62% had a past year dental visit and among those with a past year dental visit, 42% had at least a college degree. Current smokers were significantly less likely to have visited the dentist in the past year while current alcohol users were more likely than abstainers to have had a past year dental visit (Table 1). About a third (33%) self-reported ever receiving an oral cancer examination of whom 66% received this examination in the past year and 19%, 1–3 years prior. The majority (91%) of oral cancer examination was done by a dental care provider, while 9% of the examinations were done by other non-dental healthcare providers (Table 2). Being female, having at least a college degree, high household income, non-smokers, and current alcohol users were more likely than their counterparts to have ever received an oral cancer examination (Table 1).

**Table 1:** Distribution of socio-demographic factors according to time since last dental visit and oral cancer (OC) examination: NHANES 2013–2016 (*n* = 9374)

		Last dental visit ≤1 year (*n* = 5267 [62.2%])		Oral Cancer examination Yes (*n* = 2125 [33.1%])	
	Unweighted N (weighted %)	Weighted percent (SE)	*p*-value	Weighted percent (SE)	*p*-value
Age (yrs.)			0.01		< 0.0001
30- < 65	6725 (75.9)	74.5 (0.91)		70.8 (1.47)	
≥ 65	2649 (24.1)	25.5 (0.91)		29.2 (1.47)	
Age mean (95% CI)	53.1 (52.5, 53.8)	53.8 (53.1, 54.6)	0.5	55.8 (54.9, 56.8)	< 0.0001
Gender			0.001	55.0 (1.01)	0.02
Female	4917 (52.6)	54.7 (0.86)		45.0 (1.01)	
Male	4457 (47.4)	45.3 (0.86)			
Race/ethnicity			< 0.0001	1.89 (0.31)	< 0.0001
Mexican American	1376 (7.87)	5.77 (0.90)		2.04 (0.33)	
Other Hispanic	1037 (5.38)	4.61 (0.82)		84.9 (1.24)	
Non-Hispanic White	3660 (67.5)	71.6 (2.28)		6.39 (0.76)	
Non-Hispanic Black	1931 (10.8)	9.46 (1.08)		4.77 (0.46)	
Other	1370 (8.39)	8.51 (0.85)			
Education			< 0.0001	20.7 (1.32)	< 0.0001
≤ High School	4243 (35.7)	27.3 (1.63)		31.7 (1.41)	
Some college	2698 (31.3)	30.7 (1.19)		45.6 (1.97)	
≥ College	2424 (33.0)	41.9 (2.14)			
missing	9				
Household income			< 0.0001		< 0.0001
< $20 k/yr.	1835 (13.2)	8.71 (0.86)		7.32 (0.96)	
20-45 k/yr.	2757 (26.0)	21.6 (1.09)		17.7 (1.38)	
> 45 k/yr.	4203 (60.8)	69.7 (1.62)		74.9 (1.63)	
missing	579				
Smoking			< 0.0001		< 0.0001
Non-smoker	5134 (53.9)	58.9 (0.90)		56.3 (1.55)	
Current	1805 (18.7)	13.7 (0.78)		12.6 (0.99)	
Former	2424 (27.4)	27.5 (0.79)		31.1 (1.58)	
missing	11				
Alcohol consumption			< 0.0001		< 0.0001
Lifetime abstainers	1332 (14.6)	14.2 (1.61)		11.3 (1.64)	
Former drinkers	2158 (27.0)	25.0 (1.04)		23.8 (1.78)	
Non excessive current drinkers	1999 (32.9)	36.3 (1.76)		40.7 (2.12)	
Excessive current drinkers	1491 (25.5)	24.5 (1.19)		24.2 (1.83)	
missing	2394				
Smoking and alcohol use			< 0.0001		< 0.0001
Neither^a^	1183 (13.2)	13.0 (1.62)		31.8 (2.31)	
Current alcohol use only	2660 (41.0)	45.8 (1.83)		56.7 (2.56)	
Current cigarettes only	149 (1.43)	1.20 (0.13)		3.27 (0.58)	
Both	2980 (44.3)	40.0 (1.30)		8.17 (0.81)	
missing	2402				
Oral cancer examination			< 0.0001		
Yes	2125 (33.1)	44.2 (1.82)		–	
No	7249 (66.9)	55.8 (1.82)		–	
Last dental visit					< 0.0001
≤ 1 visit	5267 (62.2)	–		83.1 (1.15)	
> 1	4107 (37.8)	–		16.9 (1.15)	

^a^Refers to never and former use; p-value compares dental visit of ≤1 year to > 1 year and OC examination, yes and no

**Table 2:** Distribution selected clinical measures according to time since last oral cancer examination: NHANES 2013–2016 (*n* = 2125)

	Ever Oral Cancer screened	Most recent oral cancer screening exam^a^			*p*-value
		< 1 year (n, weighted %)	1–3 years (n, weighted %)	> 3 years (n, weighted %)	
Total	2125 (100)	1352 (66.4)	415 (18.9)	351 (14.7)	< 0.0001
	(weighted % [SE])				
Last dental visit					< 0.0001
≤ 1 year	83.1 (1.15)	77.5	14.7	7.79	
> 1 year	16.9 (1.15)	12.8	39.6	47.6	
Reason for dental visit					< 0.0001
Checkup/exam cleaning	75.6 (1.29)	71.6	17.8	10.6	
Something wrong/Pain	13.3 (1.09)	47.4	20.7	31.9	
Treatment for a previously diagnosed condition	10.3 (0.71)	55.3	25.9	18.8	
Other	0.88 (0.23)	50.9	8.31	40.8	
Type of professional					0.07
Doctor/Physician	8.61 (1.04)	72.4	27.6	–	
Nurse/NP	0.22 (0.12)	77.2	22.8	–	
Dentist (incl. Oral surgeon)	76.6 (1.40)	77.7	22.3	–	
Dental hygienist	14.0 (1.24)	83.1	16.9	–	
Other	0.61 (0.20)	58.3	41.7	–	

-did not report who performed oral cancer screening; ^a^7 missing information on when oral cancer examination was done

Current smokers in contrast to non-smokers were significantly less likely to report ever receiving an oral cancer examination, OR = 0.54 (95% CI = 0.44, 0.65; *p* < 0.001), upon covariate adjustment, this association was attenuated to null and lost statistical significance, OR = 0.94 (95% CI: 0.77, 1.15; *p* = 0.5). In contrast, former smokers when compared to non-smokers had both unadjusted OR = 1.13 (95% CI = 0.95, 1.36; *p* = 0.2) and adjusted OR = 1.14 (95% CI = 0.93, 1.38; p = 0.2) increased odds of ever receiving an oral cancer examination, although none of these estimates reached statistical significance. Current alcohol drinkers in contrast to abstainers were more likely to report ever receiving an oral cancer examination, adjusted OR = 1.26 (95% CI = 0.94, 1.69; *p* = 0.1) (Table 3).

**Table 3:** OR (95% CI) of self-report of ever receiving an oral cancer examination: NHANES 2013–2016

	Unadjusted	*p*-value	Adjusted	*p*-value
Smoking (n = 8781)^a^				
Non-smoker	ref.		ref.	
Current	0.54 (0.44, 0.65)	< 0.0001	0.94 (0.77, 1.15)	0.5
Former	1.13 (0.95, 1.36)	0.2	1.14 (0.93, 1.38)	0.2
Alcohol consumption (n = 6586)^b^				
Lifetime abstainers	ref.		ref.	
Former drinkers	1.18 (0.87, 1.60)	0.3	1.05 (0.75, 1.47)	0.7
Current drinkers	1.64 (1.24, 2.18)	0.01	1.26 (0.94, 1.69)	0.1
Smoking and alcohol (*n* = 6582)^c^				
Neither/former	ref.		ref.	
Current drinker only	1.66 (1.36, 2.02)	< 0.0001	1.25 (0.98, 1.58)	0.07
Current smoker only	0.64 (0.41, 0.98)	0.04	0.92 (0.54, 1.56)	0.7
Current smoking and alcohol	0.67 (0.51, 0.90)	0.01	1.05 (0.75, 1.47)	0.7

Adjusted for age (modeled as continuous), gender, race/ethnicity, education and income, last dental visit

Due to the high proportion of missing information for alcohol, the smoking model did not adjust for alcohol and the alcohol model did not adjust for smoking

Age was modeled as a continuous variable

^a^n corresponds to non-missing smoking and adjustment covariates

^b^n corresponds to non-missing alcohol and adjustment covariates

^c^n corresponds to non-missing smoking, alcohol and adjustment covariates

Current and former smokers were less likely than non-smokers to report a past year oral cancer examination; unadjusted OR = 0.30 (95% CI = 0.19, 0.45; *p* < 0.001) and 0.62 (95% CI = 0.44, 0.86; *p* = 0.005) respectively and adjusted OR = 0.51 (95% CI = 0.29, 0.88; *p* = 0.02) and OR = 0.74 (95% CI = 0.53, 1.04; *p* = 0.08) respectively. Contrary to findings of ever receiving a screening, current and former alcohol use when compared to abstainers was associated with lower adjusted odds of a past year oral cancer examination, OR = 0.84 (95% CI = 0.53, 1.30; 0.4) and OR = 0.50 (95% CI = 0.30, 0.83; *p* = 0.01) respectively (Table 4).

**Table 4:** OR (95% CI) of past year receipt of oral cancer examination among participants who have ever received an examination: NHANES 2013–2016

	Unadjusted	*p*-value	Adjusted	*p*-value
Smoking (*n* = 2117)^a^				
Non-smoker	ref		ref.	
Current	0.30 (0.19, 0.45)	< 0.0001	0.51 (0.29, 0.88)	0.02
Former	0.62 (0.44, 0.86)	0.005	0.74 (0.53, 1.04)	0.08
Alcohol consumption (*n* = 1590)^b^				
Lifetime abstainers	ref.		ref.	
Former drinkers	0.65 (0.40, 1.07)	0.08	0.50 (0.30, 0.83)	0.01
Current drinkers	1.07 (0.96, 1.19)	0.2	0.84 (0.53, 1.30)	0.4
Smoking and alcohol (n = 1590)^c^				
Neither/former	ref.		ref.	
Current drinker only	1.50 (1.06, 2.13)	0.02	0.36 (0.82, 2.25)	0.2
Current smoker only	0.38 (0.18, 0.81)	0.01	0.56 (0.22, 1.45)	0.2
Current smoking and alcohol	0.66 (0.42, 1.04)	0.07	0.93 (0.46, 1.88)	0.8

Adjusted for age (modeled as continuous), gender, race/ethnicity, education and income, last dental visit

Age was modeled as a continuous variable

Due to the high proportion of missing for alcohol, the smoking model did not adjust for alcohol and the alcohol model did not adjust for smoking

^a^n corresponds to non-missing smoking and adjustment covariates

^b^n corresponds to non-missing alcohol and adjustment covariates

^c^n corresponds to non-missing smoking, alcohol and adjustment covariates

**Fig. 1:**
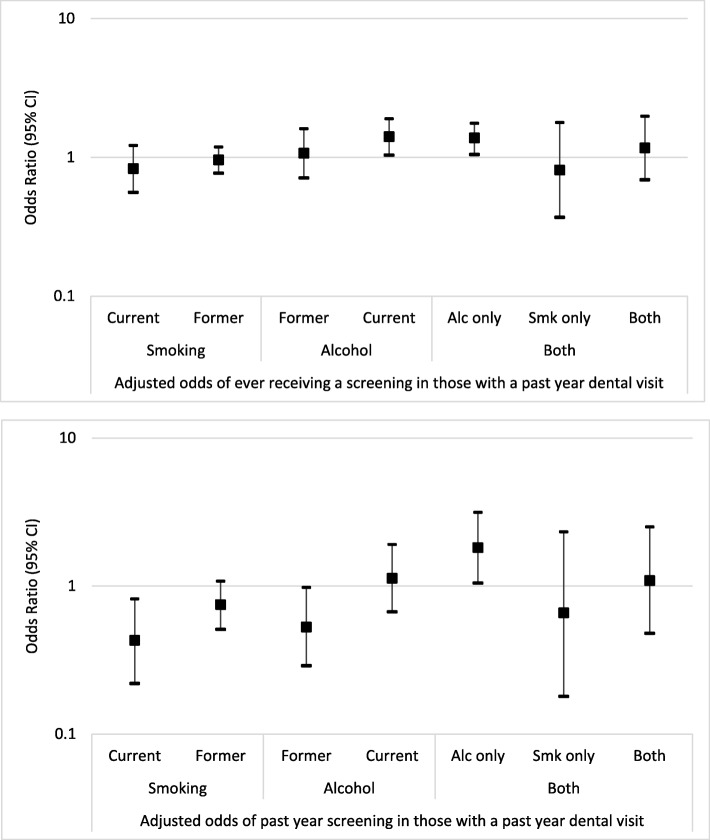
Forest plots of the natural log of the OR (95% CI) of the association between smoking, alcohol use and both with ever (Panel A) and past year (Panel B) oral cancer examination among those with a past year dental visit

The estimated odds ratios and 95% CI for reporting ever been examined for oral cancer and a past year examination among participants with a past year dental visit are presented in Fig. 1a and b respectively. The estimated ORs and 95% CIs were natural log transformed so as to be plotted on an arithmetic (with null value of zero) as opposed to a logarithmic odds ratio scale. Similar to findings for the entire study population, current alcohol use was associated with statistical increased odds of reporting ever receiving an oral cancer examination (Fig. 1a). Also similar to the entire study population, current smoking was associated with statistically lower odds of reporting a past year oral cancer examination (Fig. 1b).

## Discussion

Findings from this investigation aimed at reporting the population-level prevalence of oral cancer examination and assess if modifiable lifestyle oral cavity cancer risk factors were associated with ever and past year report of oral cancer examination, found about a third to have ever received an oral cancer examination. This estimate is higher than a 15% estimate previously reported [21] but in line with more recent estimates of 27% [22] and 30% [23]. Current smokers were less likely to have seen a dentist in the past year and also less likely to have ever or received a past year oral cancer examination. This result corroborates findings from prior studies which reported that smoking was not a significant determinant of receiving an OC examination [22, 24–26]. On the contrary current alcohol users were more likely to have had a past year dental visit and more likely to report ever been examined, similar to a previous report [22], but less likely than abstainers to have had a past year OC examination. Further, our findings indicate that conducting examinations for oral cancers is low among non-dental providers and may be an indication of the U.S. Preventive Services Task Force’s [3] conclusion of an insufficient evidence to determine the balance of benefits and harms of screening for oral cancer in asymptomatic adults in non-dental care settings. Yet, the American Head and Neck Society recommends routine examination for OC in primary care settings for individuals, especially smokers at high risk for developing OC [27].

Modifiable lifestyle related risk factors especially smoking may be a proxy for risk taking behaviors, and an indicator of being less health conscious and by extension worse dental care habits and fewer regular dental visits. Indeed, studies have reported that poor oral hygiene and a lack of regular dental visits were associated with increased odds of head and neck cancers [28–31]. Furthermore, populations deemed high risk for head and neck cancers are less likely to self-refer [11], which may partly explain the lower odds of oral cancer examination among current smokers in this study. Nonetheless, results of the sub-analysis restricted to those with a past year dental visit were consistent with those for the entire study population and suggest that factors beyond access to care or attendance of routine dental visits is contributing to the lower odds of oral cancer examination among high risk individuals, especially current smokers. Indeed, our findings suggest that oral cancer examination may not be routinely performed among patients who attend dentist offices, including among high risk individuals who smoke and use alcohol. Internal factors including low level knowledge and confidence about oral cancer and external clinic factors especially time were reported by dental professionals as barriers to screening high-risk patients [32–35]. Providing oral cancer screening continuing education (CE) opportunities to dental professionals may be necessary to address this barrier. A study conducted by Walsh et al., found OC screening CE to significantly influence dental hygienist’s knowledge and behaviors about OC screening and tobacco cessation [36]. Further, focus groups with dentists revealed a desire to improve their OC screening habits and provider-patient communication skills [37].

Oral cancer screening and symptom recognition is a preventative approach that may be a cost effective approach for the outcomes and healthcare costs of high-risk individuals as opposed to a curative model of higher healthcare and treatment costs [38, 39]. Nonetheless screening is not without challenges, including monetary and emotional costs associated with a false-positive finding, false-negative finding, failure to prevent cancer, and cancer development in-between screenings [11]. While OC screening is recommended for high risk patients in the dental office, evidence to support population-based screening efforts is lacking. Specifically, the majority of people who respond to oral cancer screening calls are a select group, most of whom do not have traditional oral cancer risk factors (smoking, alcohol and low SES) [40–42]. Therefore, targeted screening and follow-up in high risk groups that can easily be accomplished in the dentists’ office may be ideal for oral cancer screenings.

Yet, solely increasing the number of dentists who perform OC screening is insufficient to improve the OC screening rates among smokers. Indeed, participants who reported a past year dental visit were more likely to be college educated, have a high SES and be non-Hispanic white, which highlights the existing socioeconomic and racial disparities in access to dental care. In fact, smokers tend to have lower SES and education, non-private insurance and are less likely than their counterparts with private insurance to report OC screening by a health care professional [23]. Furthermore, a significantly lower percentage (53%) of Americans had dental insurance in 2008 as compared to 89% with medical insurance [43, 44]. Moreover, 70% of smokers reported an outpatient physician visit in the past year [45] and thus suggest that physicians tend to be the first point of contact for smokers. Hence, improving OC screening rates among smokers requires that targeted examinations are also conducted within primary care settings, as emphasized by Macpherson et al. [46], as well as incorporating opportunistic OC screenings into routine physical examinations of minority populations as suggested by Oh et al., [47].

### Implications for clinical practice and education

Results of studies conducted in the U.K. and U.S. regarding attitudes to screening for OC among primary care providers indicate that the low screening rates in primary care settings may be a result of a lack of understanding of the epidemiology and natural history of the disease, low confidence in providing screening and concerns about false positives, a lack of proper training, time and equipment as well as the notion that screening is within the purview of the dental care provider [48–50]. Educational initiatives including oral cancer screening continuing education programs may be needed to improve literacy surrounding oral cancer risk factors among healthcare providers and patients. Furthermore, implementation of theory-based interventions may be necessary to improve general medical practitioner’s confidence, expertise and knowledge in conducting opportunistic OC screenings [51] as well as exposure to OC prevention and detection as part of the medical school’s curriculum [52]. Lastly, the use of evidence-based screening, brief intervention and referral (SBIRT) for tobacco and alcohol-use disorders, previously shown as effective in dental practice [53, 54] can be helpful in identifying high risk individuals for OC screening.

### Strengths and limitations

Some of our study’s limitations include the self-reported nature of our measures including oral cancer examination, which is subject to misreporting and recall bias. If there is non-differential reporting of oral cancer examination among the exposure groups, then our reported measures of associations are likely biased towards the null. With differential reporting however, the direction of bias is hard to predict. The oral cancer examination question did not differentiate between opportunistic screening and screening in response to mucosal complaint, as these groups may be systematically different.

Misclassification of smoking and alcohol use is also likely given the self-reported nature of these measures. Nevertheless, self-reported smoking status has been shown to correlate well with serum cotinine levels [55].

Next, the cycles of NHANES used for this study did not include questions on OC risk perception, knowledge and beliefs as studies have shown that OC risk perception vary by risk behaviors [56] and that fear may be a barrier to participation in OC screening [57]. This is an area that should be further explored in future studies. Other potential predictors of OC examination such as distance and location of healthcare facility as well as dental insurance status are not available in the NHANES public use datafiles as adjustment variables. Despite these limitations, a major strength of this study is that findings were based on a large nationally representative sample of U.S. adults, making our results less subject to random error. Another of this study’s strength is that it focuses on the modifiable lifestyle oral cavity cancer risk factors (smoking and alcohol consumption) as well as assesses the joint effects of use of both alcohol and cigarettes.

## Conclusions

Current smokers were less likely than non-smokers to have seen a dentist in the past year and also less likely than non-smokers to have ever or received a past year oral cancer examination while current alcohol users were more likely than abstainers to have had a past year dental visit and more likely to report ever been examined for OC but less likely than abstainers to have had a past year OC examination. Given that the majority of oral cancer screenings occur in dental settings, it suffices to say that smoking more so than alcohol use likely represent proxies for dental care seeking behaviors, whereby current smokers are less likely to attend routine dental visits and by extension fewer opportunities for oral cancer examination. In the same vein, dental visits among smokers and alcohol consumers does not guarantee receipt of an oral cancer examination. Thus, this represents both an issue of access as well as a need to educate and further reiterate the necessity to screen patients in the dental care setting and to introduce opportunistic OC screenings for high risk individuals into primary care settings.

## Data Availability

The datasets generated and/or analyzed during the current study are available in the NHANES repository, https://wwwn.cdc.gov/nchs/nhanes/Default.aspx

## Abbreviations

ADA: American Dental Association
CI: Confidence Interval
HPV: Human Papilloma Virus
NHANES: National Health and Nutrition Examination Survey
OC: Oral Cancer
OR: Odds Ratio
U.S.: United States
